# Early detection of Parkinson’s disease through enriching the electronic health record using a biomedical knowledge graph

**DOI:** 10.3389/fmed.2023.1081087

**Published:** 2023-05-12

**Authors:** Karthik Soman, Charlotte A. Nelson, Gabriel Cerono, Samuel M. Goldman, Sergio E. Baranzini, Ethan G. Brown

**Affiliations:** ^1^Department of Neurology, Weill Institute for Neurosciences, University of California, San Francisco, San Francisco, CA, United States; ^2^Division of Occupational and Environmental Medicine, University of California, San Francisco, San Francisco, CA, United States

**Keywords:** Parkinson disease, neurodegenerative disorder, electronic health record, knowledge graph, graph algorithm, machine learning

## Abstract

**Introduction:**

Early diagnosis of Parkinson’s disease (PD) is important to identify treatments to slow neurodegeneration. People who develop PD often have symptoms before the disease manifests and may be coded as diagnoses in the electronic health record (EHR).

**Methods:**

To predict PD diagnosis, we embedded EHR data of patients onto a biomedical knowledge graph called Scalable Precision medicine Open Knowledge Engine (SPOKE) and created patient embedding vectors. We trained and validated a classifier using these vectors from 3,004 PD patients, restricting records to 1, 3, and 5 years before diagnosis, and 457,197 non-PD group.

**Results:**

The classifier predicted PD diagnosis with moderate accuracy (AUC = 0.77 ± 0.06, 0.74 ± 0.05, 0.72 ± 0.05 at 1, 3, and 5 years) and performed better than other benchmark methods. Nodes in the SPOKE graph, among cases, revealed novel associations, while SPOKE patient vectors revealed the basis for individual risk classification.

**Discussion:**

The proposed method was able to explain the clinical predictions using the knowledge graph, thereby making the predictions clinically interpretable. Through enriching EHR data with biomedical associations, SPOKE may be a cost-efficient and personalized way to predict PD diagnosis years before its occurrence.

## 1. Introduction

Parkinson’s disease (PD) is a progressive neurodegenerative condition that affects 2–3% of people over 65 years old (1) and is the most rapidly increasing neurological disorder worldwide (2). To date, no intervention has been proven to slow disease progression in PD (3). A major barrier to discovering effective therapies may be that patients are not diagnosed with PD until motor symptoms, such as tremor and bradykinesia, manifest (4). But these symptoms only arise after ~50% of the neurons in the substantia nigra, the main brainstem area affected in PD, have already been lost (5). Diagnosing people earlier (i.e., before they develop frank motor symptoms), has been proposed as a necessary step to effective testing and implementation of disease-modifying treatments (6).

A window of opportunity to diagnose people with PD earlier lies in the prodromal stage: a period of time prior to development of motor symptoms when early pathological changes lead to numerous other symptoms, such as autonomic, sleep, and mood problems (7). These symptoms bring people to the attention of physicians and are coded as diagnoses in the electronic health record (EHR), raising the possibility that the medical chart can be used to identify people in this early stage. While a single diagnosis may be common in an older population and not specific, the presence of multiple relevant diagnoses simultaneously can be used to identify people who are at risk of developing PD (8, 9). Indeed, algorithms that combine information from the EHR have been reported to help identify people at risk of PD (10–12). However, these models have largely been driven by motor conditions, indicating that a patient may already have PD and substantial central neurodegeneration. Patients likely meet diagnostic criteria for PD well before a code appears in the medical record, leading to a median delay of around 1 year between the presence of PD and the recording in the EHR (13). Constructing predictive models based on codes that are present years prior to the appearance of a PD diagnostic code could further the utility of these models for targeting patients that may benefit from interventions. Additionally, broadening the EHR variables incorporated into the model beyond diagnoses may improve their predictive power and allow for discovery of novel biomedical relationships.

In this project, we applied machine learning (ML) techniques to diagnosis, medication, and laboratory codes from the de-identified EHR data of the University of California San Francisco Medical Center (UCSF) to determine whether a diagnosis of PD could be predicted years before the clinical diagnosis. Because these EHR codes are primarily used for billing purposes, a hypothesis-free ML may generate spurious results that reflect coding habits or practices specific to an institution that are less likely to be applicable to other practice settings and are not biologically meaningful (14). To bring meaningful biological associations into the context, we mapped these EHR concepts onto a heterogeneous biomedical knowledge network - the Scalable Precision medicine Oriented Knowledge Engine (SPOKE) - that combines over 30 biologically relevant public databases and describes meaningful associations between nodes such as disease, genes, drugs, protein etc., (15, 16). We hypothesized that incorporation of such biomedical associations could enrich the clinical data and aid in identifying people with PD years before the actual diagnosis arose in the EHR.

## 2. Materials and methods

### 2.1. Patient selection

**Figure 1:**
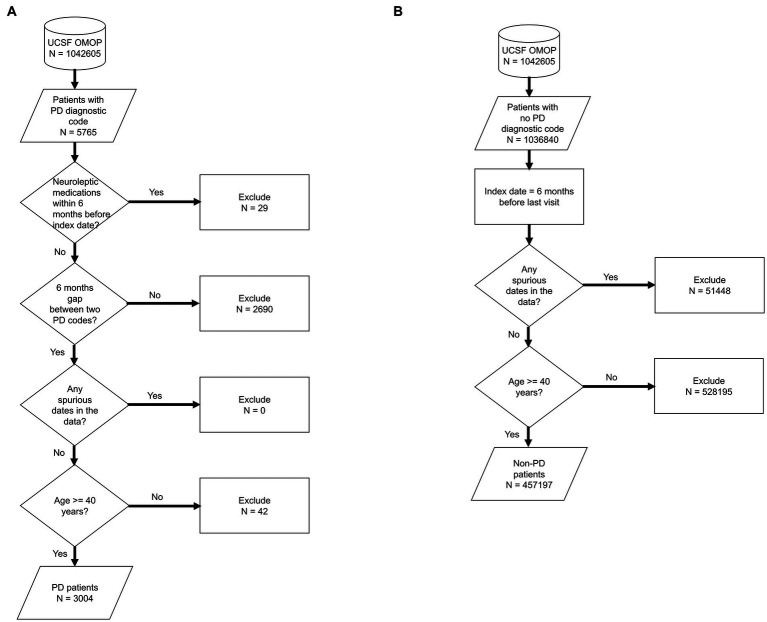
Flowchart of population selection for PD **(A)** and non-PD **(B)** cohort. It starts from UCSF OMOP database followed by criteria for patient selection, number of patients included/excluded based on the selection criteria at each stage. Each shape in the diagram corresponds to the standard flowchart symbol. Cylindrical shape depicts a database. Diamond shape depicts a decision/criteria which can have yes and no branches. Parallelogram shape depicts an input or output of a process. Rectangle shape depicts a process or action that is taken based on a criteria or input data.

**Figure 2:**
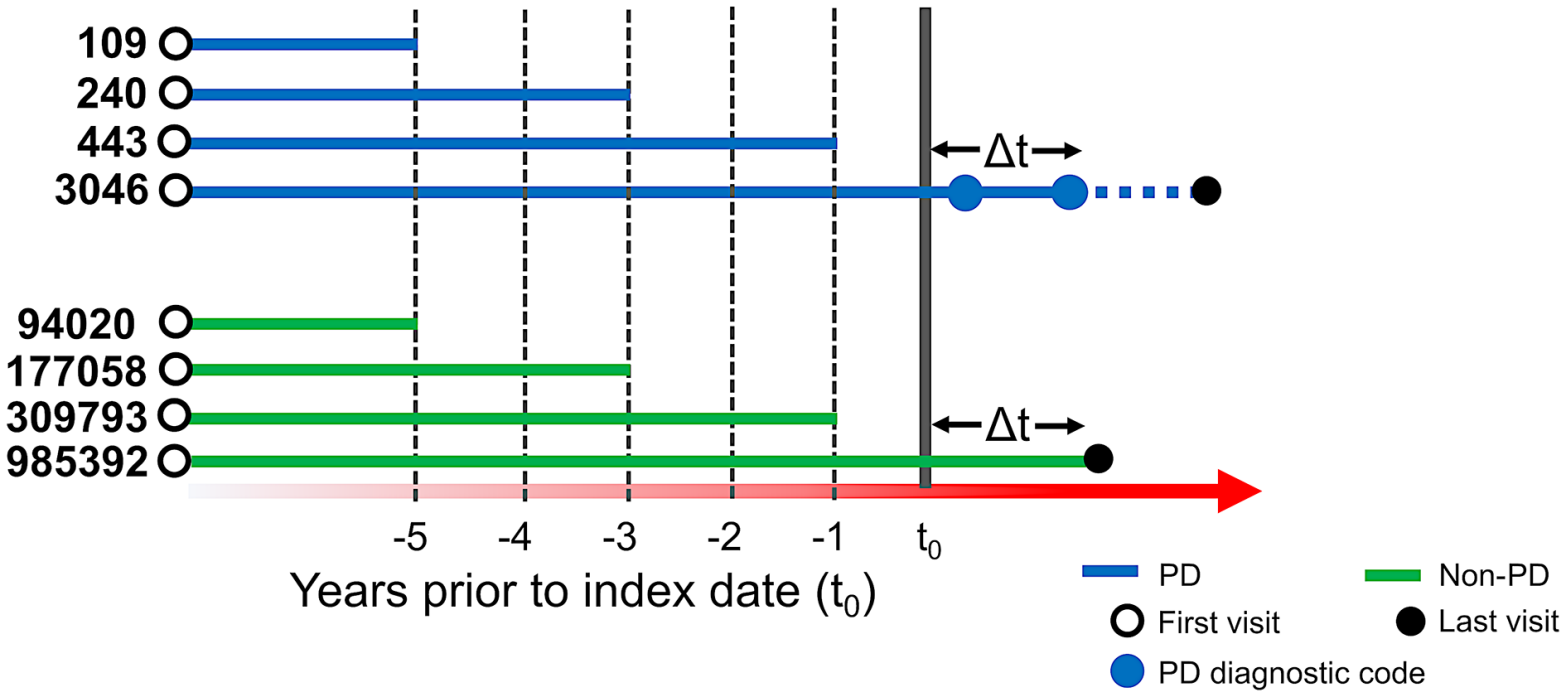
Schema of time periods selected for analysis. t_0_ represents the index date. For people with PD (blue lines), t_0_ is defined as the first PD-related diagnostic code in the electronic health record (EHR) or first use of PD-related medication. Solid blue dots represent subsequent diagnostic codes of PD and the solid black dot represents the last visit in the EHR. For people with PD, at least two diagnostic codes were required, and the difference between t_0_ and another diagnostic code (△t) had to be at least 6 months. For people without PD (green lines), t_0_ was set to 6 months (△t) before their last visit (solid black dot). For our predictive models, we conducted three separate analyses, restricting EHR data to anything present before 1, 3, and 5 years prior to index date. The number of patients in each analysis group is provided to the left of each line (prior to application of the 40 year old age threshold).

We used de-identified EHR data of patients who visited UCSF between 2010 and 2020. Patient cohort selection was performed based on the protocol described in (16) (Supplementary methods). Two patient cohorts (i.e., PD and non-PD) were created based on the presence of diagnostic codes indicative of PD in their EHR diagnosis table (Figure 1; Supplementary Table S1). To avoid inclusion of patients with neuroleptic-induced parkinsonism, a common misdiagnosis, patients on neuroleptic medications (Supplementary Table S2) within 6 months before their first PD diagnosis were excluded (Figure 1A). We restricted the entire population to 40 years of age or older, to minimize the inclusion of people with rare genetic forms of PD who may have patterns of onset different than sporadic PD. Implementing this age criteria also avoids overrepresentation of younger controls, which would lead to conditions associated with aging appearing to be associated with PD development. The index date for PD was defined as the first entry of a PD code or, for patients started on medications for PD (Supplementary Table S3) prior to the appearance of the EHR code, the date this medication was started. In order to build a classifier that would identify people at risk of PD in the general population, we trained the model for each time period using a case:control ratio based on the age-adjusted prevalence of PD, i.e., 572:100,000 among people of age 45 and over (17), which closely matches the age threshold in this study. Further, we categorized the EHR data of selected patient cohorts into three pre-diagnostic time periods that included data present one (−1), three (−3) and five (−5) years prior to their index date (Figure 2; Supplementary methods).

### 2.2. Patient embedding vectors

**Figure 3:**
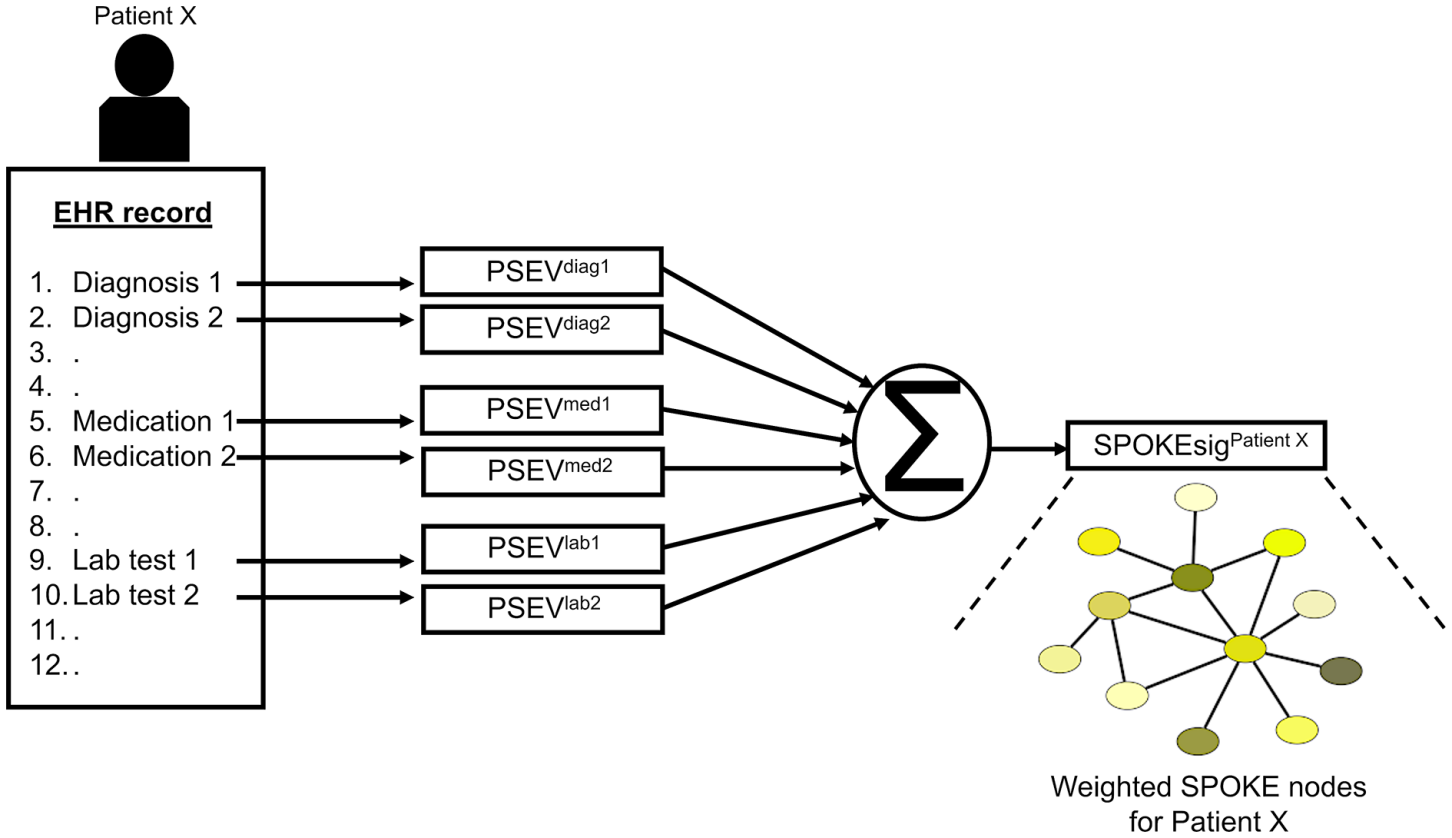
Schema of patient SPOKEsig generation. Diagnostic codes, prescribed medications, and laboratory test values are taken from the electronic health record (EHR, left side of image) and translated into codes readable by SPOKE. Integration of these concepts into SPOKE creates Propagated SPOKE Entry Vectors (PSEVs), which are then added together to create the SPOKEsig vector for each patient. The SPOKEsig of a patient represents a network of SPOKE nodes (right side of the figure; color represents node type), where each SPOKEsig value represents the relevance of the corresponding node in the network for that patient. Therefore, an element (or feature) in the SPOKEsig vector corresponds to a node in the SPOKE knowledge graph and the value depicts the weight of that node.

After patient selection, we created knowledge graph (SPOKE) based embedding vectors for these patients (15, 16). This was achieved by connecting EHR concepts (diagnosis, medication and lab test) to nodes in the SPOKE knowledge graph using Unified Medical Language System’s (UMLS) Metathesaurus mappings. After making these connections, as previously described in (15), a modified version of topic-sensitive PageRank algorithm (18) was implemented to generate a vector that describes importance of each node in the graph relative to the EHR variable of interest. This vector was called as Propagate SPOKE Entry Vector (PSEV) (15). PSEV can be treated as a network level embedding vector for a clinical concept and it can be created for any EHR concept corresponding to a cohort of patients (for, e.g., Parkinson’s disease). To create embedding at an individual patient level, PSEVs corresponding to the EHR variables of a patient are added and normalized (16) (Figure 3; Supplementary methods). Each element in the resulting vector corresponded to a SPOKE node and determined the relevance of that node for the patient. Hence, we called the resulting representation as patient SPOKEsig (short for SPOKE signature) (16).

### 2.3. SPOKEsig feature analysis

To explore the individual features of SPOKEsig representations, we compared each feature node between PD and non-PD cohorts for each time period. We used Mann–Whitney U rank test to compare the distributions of values at each SPOKE feature node. We then repeated this analysis for all three time periods to determine how this comparison changed across the pre-diagnostic time frame.

### 2.4. Training and testing of random forest classifier

Random forest classifier was used to classify patients as PD or non-PD in each time period. Patient SPOKEsigs in a time period were first split into train and test datasets in 80:20 ratio, respectively. Training data was used to train the classifier and testing data was used to evaluate the performance of the classifier. To reduce the bias from an imbalanced dataset (i.e., more non-PD samples than PD samples) while training the classifier, PD samples were weighted more heavily than the non-PD samples based on their distribution in the training data of a given time period. Classifiers were trained in an online batch wise fashion and by using parallelization to optimize memory and training time, respectively. Area under the curve (AUC) was used as the performance metric of the classifier. The testing phase of the model, after training, was done by bootstrapping the test data. Bootstrapping was done by running model predictions 100 times, each time on a randomly selected patient set (with 50 patients including both classes) with replacement from the test dataset. This generated a set of ROC curves and a distribution of AUC scores for the model in each time period. A 95% bootstrap confidence interval (CI) was then computed by taking the 2.5th and 97.5th percentiles of the AUC distribution for each time period. Finally, we compared the performance of the random forest classifier with a logistic regression model to account for any algorithm-specific differences in predicting PD using SPOKEsig vectors (see Supplementary methods).

### 2.5. Comparative analysis

#### 2.5.1. Raw EHR data

For comparative analysis, we performed predictions for PD using raw EHR data (i.e., without SPOKE enrichment). We created binary representation vectors of patients using their EHR chart from each time period (Supplementary methods). For a fair comparison with SPOKE, we restricted EHR concepts to those mappable to SPOKE nodes (Supplementary Table S8). We further trained a random forest classifier with these raw EHR representations and compared its predictive performance with the SPOKE method (Supplementary methods).

#### 2.5.2. MDS criteria

SPOKE-based prediction results were compared with analysis of EHR data according to the proposed research criteria for prodromal PD developed by the International Parkinson and Movement Disorder Society (MDS) (8, 9). The MDS method estimates a likelihood ratio for future PD diagnosis based on the presence or absence of numerous risk and prodromal markers that are supported by the literature. Using the likelihood ratio and prior probability (based on patient’s age) we then computed the patient’s posterior probability for prodromal PD. This prediction was further compared with the SPOKE method using AUC bootstrap analysis (Supplementary methods).

#### 2.5.3. Clinician review

SPOKE-based prediction results were also compared with the review of de-identified EHR data by a movement disorders neurologist specialized in the diagnosis and therapeutics of PD and other movement related disorders. The neurologist reviewed the EHR chart of hundred unique patients in each time period and classified them as either prodromal PD or not (Supplementary methods). These predictions were further compared with the SPOKE based predictions for the same patients using AUC bootstrap analysis (Supplementary methods).

## 3. Results

### 3.1. Patient data

We identified 3,046 patients with a diagnosis of PD (Figure 1A) and then selected 985,392 individuals without any diagnosis of PD (Figure 1B). We then restricted this population to only include patients who were at least 40 years old (*n* = 3,004 for PD and *n* = 457,197 for non-PD, Figure 1). Finally, as people may meet criteria for PD before it is coded in the EHR (13) and we sought to target a prodromal population, we restricted our analysis to EHR information one, three, and 5 years prior to the appearance of the first PD-related diagnostic code or medication (referred to as −1, −3, and −5 time periods; Figure 2; Supplementary Tables S4, S5).

### 3.2. SPOKEsig feature analysis

**Figure 4:**
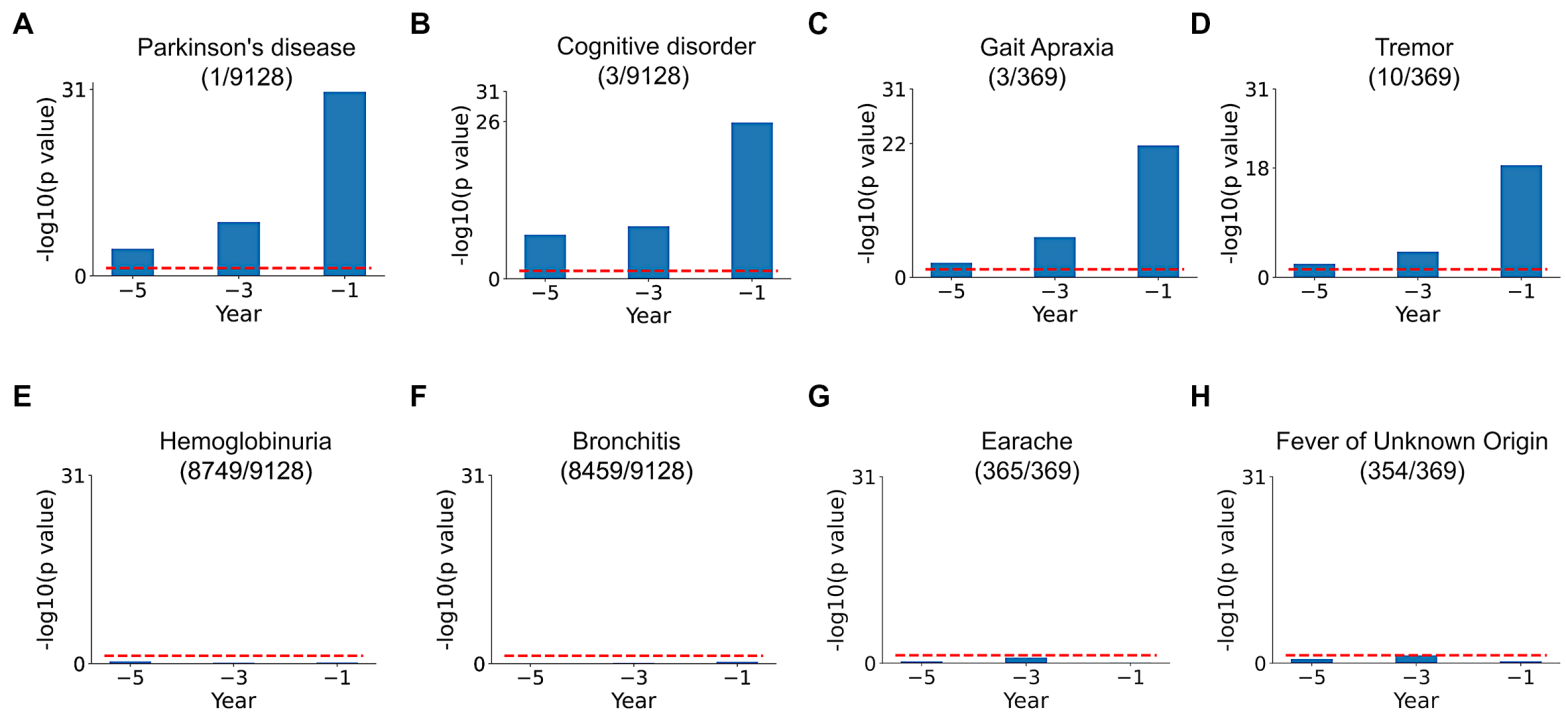
SPOKEsig feature analysis. Examples shown include disease and symptom features related **(A–D)** and not related **(E–H)** to PD. Blue bars represent feature significance between PD versus non-PD populations across pre-diagnosis time periods (x-axis). The red dotted line shows the threshold level of significance (*p* = 0.05). A rank is assigned to features based on their significance values (shown in parentheses as rank/total count of that feature in SPOKE).

Comparison of SPOKEsigs between PD and non-PD cohorts revealed a number of relevant differences (Figures 4A–D). Despite not being explicitly coded in the EHR, the PD node (i.e., the biomedical concept from SPOKE knowledge graph) had a significantly higher value in the PD population compared to non-PD population across all three time periods (Figure 4A). Additionally, other related disease (i.e., Cognitive disorder, Figure 4B) and symptom (i.e., Tremor and Gait Apraxia, Figures 4C,D) nodes showed higher values in PD compared to non-PD groups. On the other hand, values for disease and symptom nodes not related to PD were not significantly different between the two cohorts (Figures 4E–H).

### 3.3. Patient classification using random forest classifier

**Figure 5:**
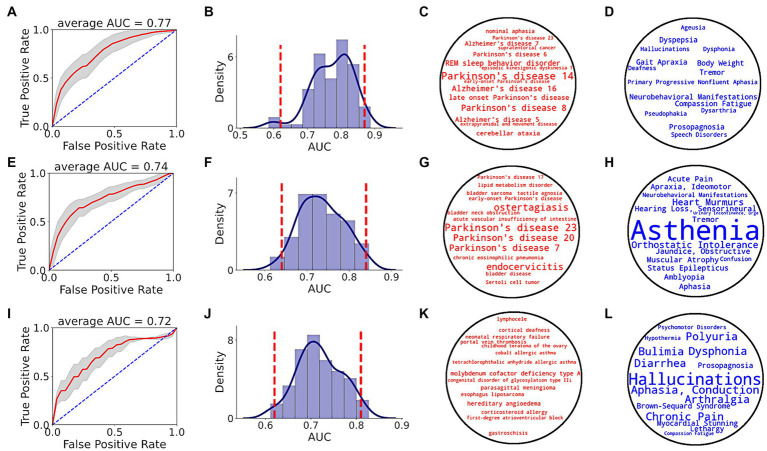
PD prediction using SPOKE based model. Each row in the figure corresponds to a single time period, i.e., EHR data from at least 1 **(A–D)**, 3 **(E–H)** and 5 **(I–L)** years prior to index date. **(A,E,I)** show ROC performance curves of random forest classifiers across respective time periods, with red curve depicting the average ROC curve, gray shade showing ± standard deviation, and blue dotted line representing the random guess curve. Average AUC of each ROC curve is shown at the top of each box. **(B,F,J)** show AUC distributions of classifiers across respective time periods. Vertical red dotted lines indicate 95% confidence interval of each AUC distribution. **(C,G,K)** show top 15 disease nodes, and **(D,H,L)** show top 15 symptom nodes, for patient classification across respective time periods, with word size proportional to feature importance.

Prediction performance of random forest classifier, i.e., AUC score, to distinguish between PD and non-PD patients based on pre-diagnostic data of each time period is shown in Figure 5. Average AUC scores of the classifiers increased from −5 to −3 and −1 time periods (Table 1).

**Table 1:** Classifier AUC performance across pre-diagnosis time periods.

Year	AUC (μ ± σ)	95% bootstrap confidence interval
−1	0.77 ± 0.06	(0.62, 0.87)
−3	0.74 ± 0.05	(0.64, 0.84)
−5	0.72 ± 0.05	(0.62, 0.81)

Analyzing the top input feature nodes, we found that several nodes related to PD were among the top 15 disease (Figures 5C,G,K) and symptom (Figures 5D,H,L) nodes in the periods closer to the index date, even though the PD diagnosis was not present in the medical record. For example, in the −1 period, nodes with high predictive power included various types of PD (as described in the Disease Ontology), REM sleep behavior disorder (a condition that is highly predictive of PD), tremor and gait apraxia (symptoms common in PD; Figures 5C,D). Unlike periods −1 and −3, no explicit PD nodes were identified for period−5 (Figure 5K), though several symptoms relevant to the pre-diagnostic stages of PD were identified (e.g., dysphonia, polyuria, chronic pain, lethargy; Figure 5L). In addition to clinical feature nodes like disease and symptom, several gene nodes related to PD appeared in the top tier (>90th percentile of feature score distribution). Genes related to PD such as GBA (99.2 percentile score), LRRK2 (98.6 percentile score), PINK1 (97.3 percentile score), ATP13A2 (97.2 percentile score), VPS35 (96.3 percentile score), and PARK7 (94 percentile score) served as critical features for the classifier in detecting prodromal PD patients in −1 time period (see Supplementary Table S7 for a list of top biological nodes across all time periods). Taken together, these results highlight the increasing flow of PD-related information in the SPOKE embeddings of PD patients as time to their diagnosis approaches.

We also compared the predictive performance of random forest classifier with a logistic regression model using the same patient test data at −1 time period. We found that predictive performances of both classifiers were not significantly different on the given test data (random forest AUC = 0.77 ± 0.06, logistic regression AUC = 0.77 ± 0.062, Kolmogorov–Smirnov test value of *p* = 0.97, Kolmogorov–Smirnov statistic = 0.07, *N* = 100, Supplementary Figure S1).

### 3.4. Comparative analysis

#### 3.4.1. Raw EHR data

**Figure 6:**
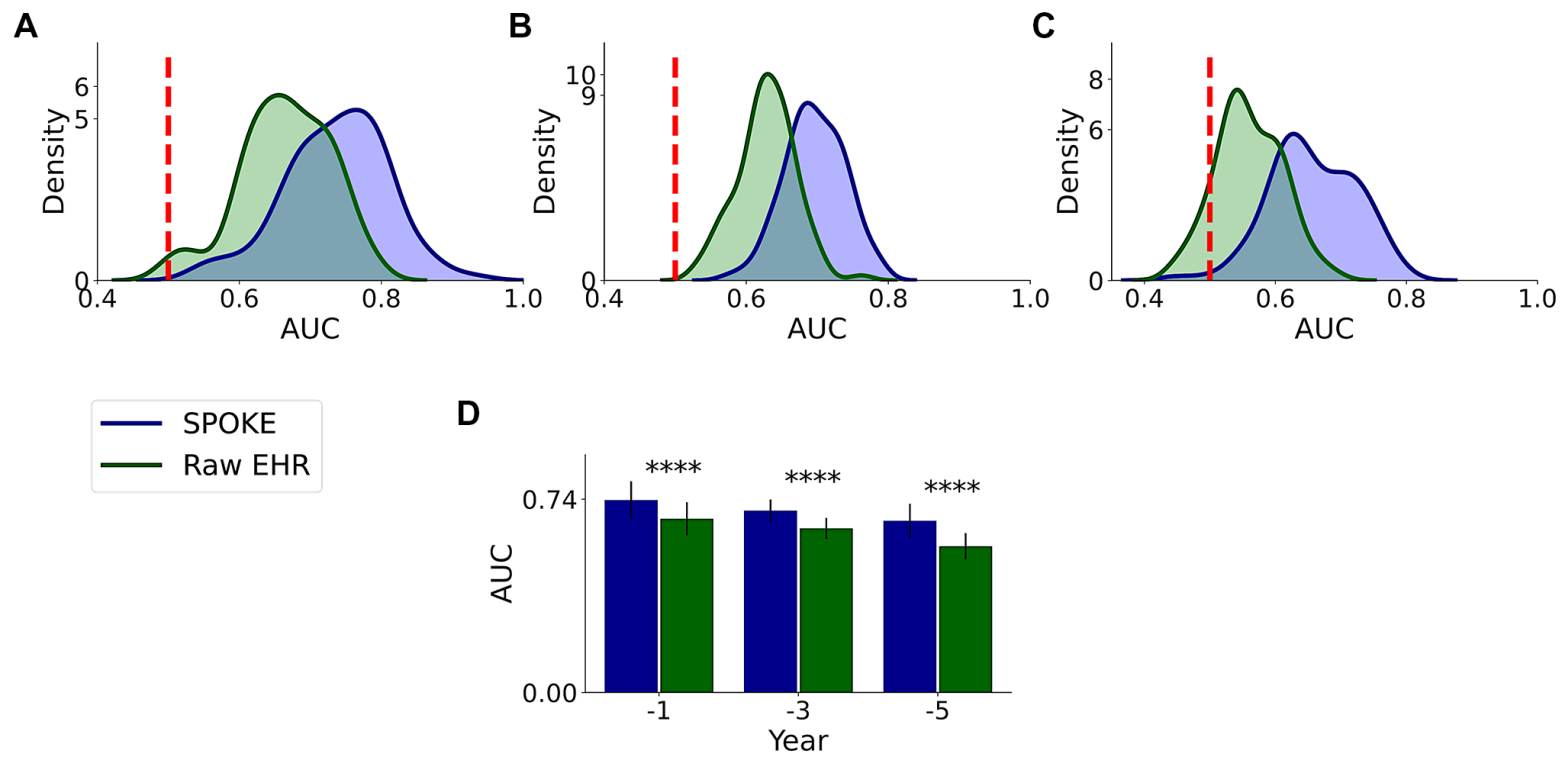
Comparative analysis of PD prediction between SPOKE and raw EHR data. Distributions of classification AUC scores between SPOKE (blue) and raw EHR (green) across −1, −3 and −5 year time periods are shown in **(A–C)** respectively. Vertical red dashed line indicates an AUC score of 0.5 which corresponds to random guessing. **(D)** shows a bar graph with mean and standard deviation of AUC distributions for SPOKE (blue) and raw EHR (green) across time periods shown along the x-axis. Asterisks in the graph indicate *p* value significance of ≤0.0001.

We compared the performance of both raw EHR and SPOKE-based classifications and found that across all three time periods, SPOKE-based classifier was more accurate than a classifier limited to raw EHR data in predicting PD diagnosis (Figure 6; Table 2).

**Table 2:** SPOKE versus raw EHR performance comparison across pre-diagnosis time periods.

Year	SPOKE AUC (μ ± σ)	raw EHR AUC (μ ± σ)	*p* value (*t*-test, *N* = 100)	SPOKE AUC 95% CI	raw EHR AUC 95% CI
−1	0.74 ± 0.07	0.67 ± 0.06	3.4*10^−12^	(0.57, 0.86)	(0.51, 0.78)
−3	0.7 ± 0.04	0.63 ± 0.04	2.4*10^−24^	(0.61, 0.78)	(0.55, 0.7)
−5	0.66 ± 0.07	0.56 ± 0.05	1.5*10^−24^	(0.54, 0.77)	(0.46, 0.66)

#### 3.4.2. MDS criteria

**Figure 7:**
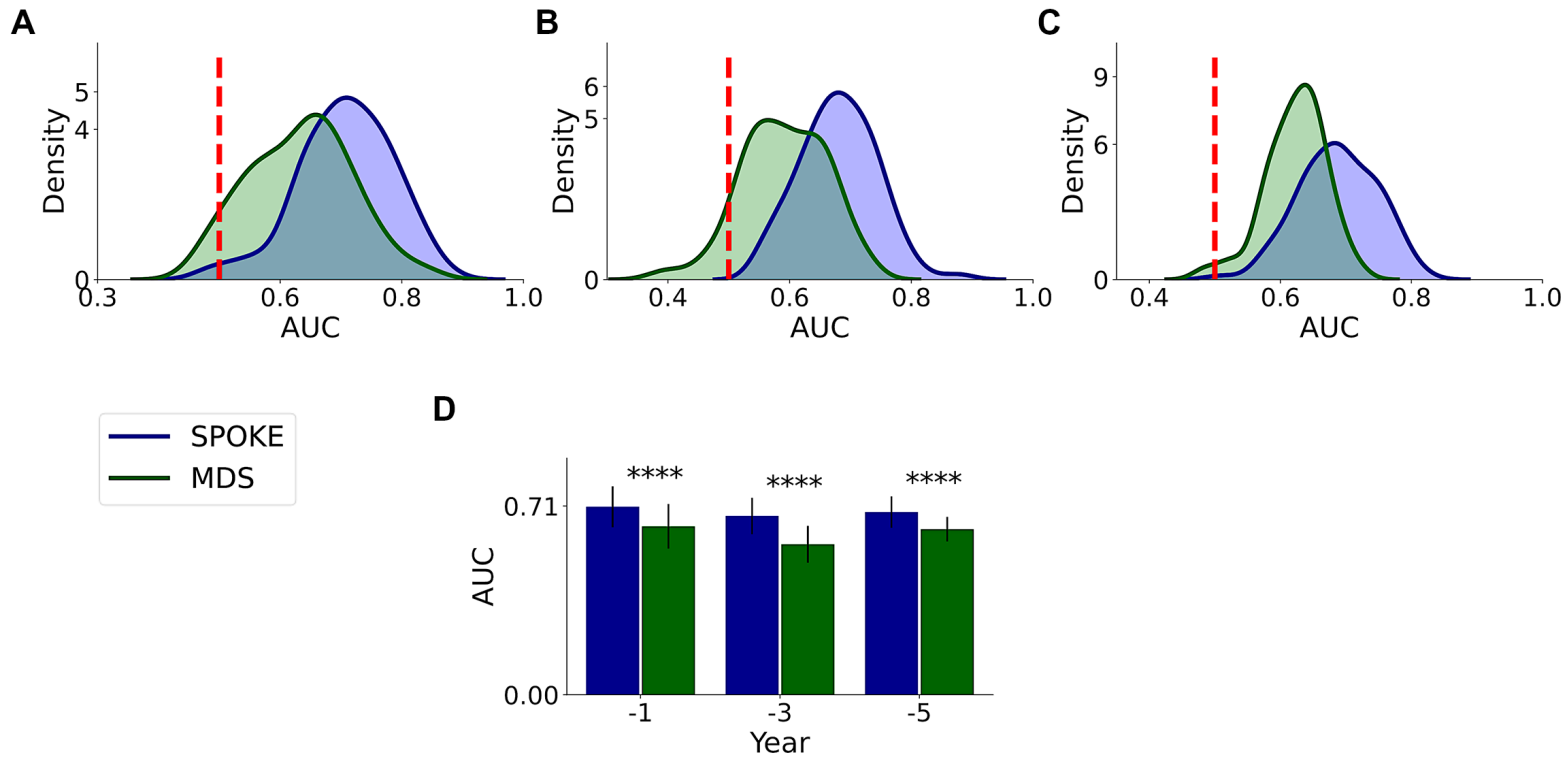
Comparative analysis of PD prediction between SPOKE and MDS criteria. Distributions of classification AUC scores between SPOKE (blue) and MDS prodromal criteria (green) across −1, −3 and −5 year time periods are shown in **(A–C)** respectively. Vertical red dashed line indicates an AUC score of 0.5 which corresponds to random guessing. **(D)** shows a bar graph with mean and standard deviation of AUC distributions for SPOKE (blue) and MDS (green) across time periods shown along the x-axis. Asterisks in the graph indicate p value significance of ≤0.0001.

This comparative analysis was done on 37,233, 21,730 and 11,299 unique patients with MDS markers among our originally selected cohort in −1, −3 and −5 periods, respectively, (Supplementary methods). We found that SPOKE performance was higher than MDS criteria using EHR data in predicting PD across all three time periods (Figure 7; Table 3).

**Table 3:** SPOKE versus MDS performance comparison across pre-diagnosis time periods.

Year	SPOKE AUC (μ ± σ)	MDS AUC (μ ± σ)	*p* value (*t*-test, *N* = 100)	SPOKE AUC 95% CI	MDS AUC 95% CI
−1	0.71 ± 0.08	0.63 ± 0.08	8.5*10^−10^	(0.53, 0.84)	(0.49, 0.79)
−3	0.67 ± 0.07	0.57 ± 0.07	5.1*10^−22^	(0.56, 0.79)	(0.46, 0.72)
−5	0.69 ± 0.06	0.62 ± 0.05	4.1*10^−15^	(0.58, 0.79)	(0.52, 0.7)

#### 3.4.3. Clinician review

**Figure 8:**
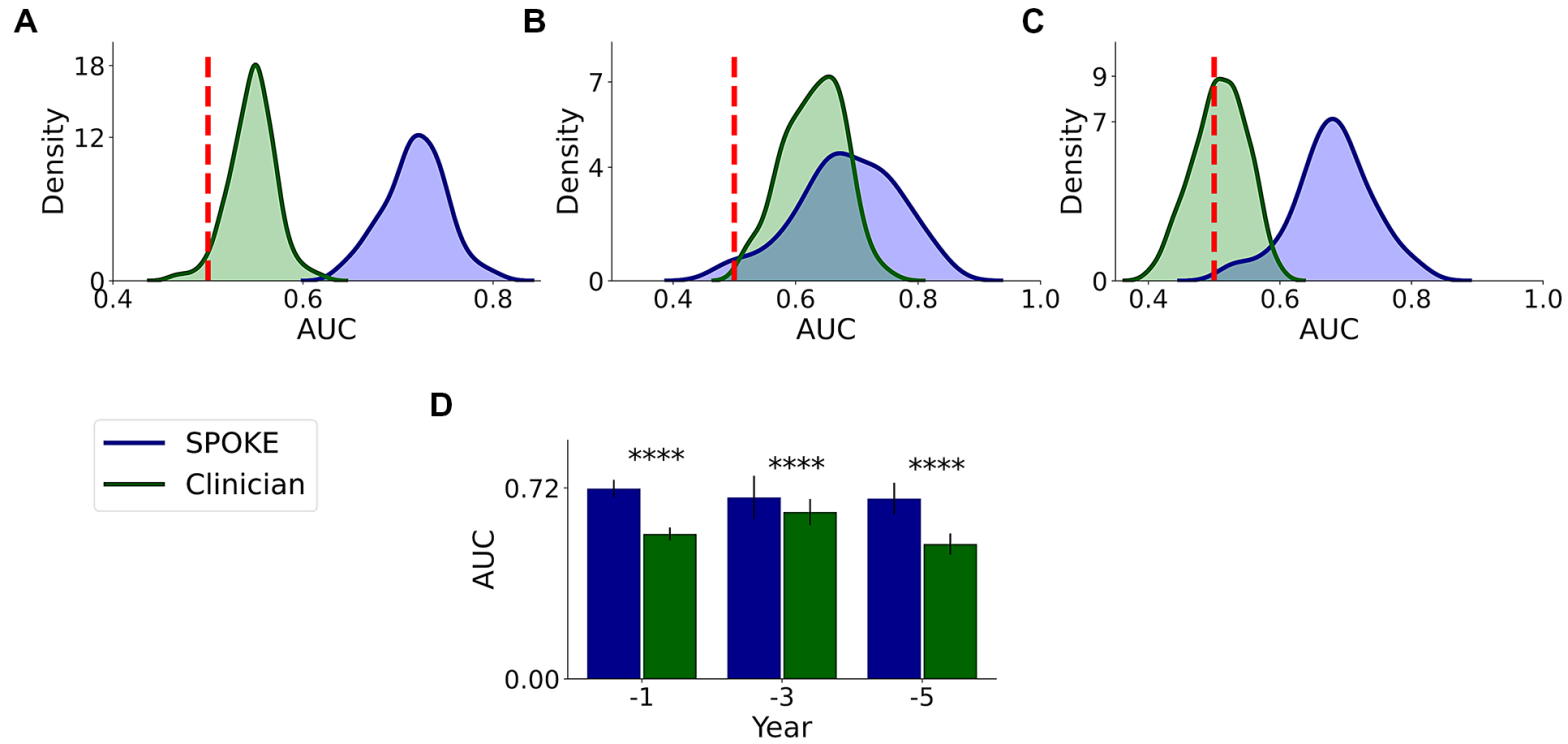
Comparative analysis of PD prediction between SPOKE and clinician review of EHR data. Distributions of classification AUC scores between SPOKE (blue) and clinician review (green) across −1, −3 and −5 year time periods are shown in **(A–C)** respectively. Vertical red dashed line indicates an AUC score of 0.5 which corresponds to random guessing. **(D)** shows a bar graph with mean and standard deviation of AUC distributions for SPOKE (blue) and clinician (green) across time periods shown along the x-axis. Asterisks in the graph indicate p value significance of ≤0.0001.

We had a movement disorders clinician (EGB) review the EHR data of patients to which SPOKE had access and predict if the patients would be diagnosed with PD or not (Methods and Supplementary methods). Comparative analysis showed that SPOKE method had higher prediction performance than clinician review of the EHR data in predicting which patients would develop PD using pre-diagnosis data across all three time periods (Figure 8; Table 4).

**Table 4:** SPOKE versus clinician performance comparison across pre-diagnosis time periods.

Year	SPOKE AUC (μ ± σ)	Clinician AUC (μ ± σ)	*p* value (*t*-test, *N* = 100)	SPOKE AUC 95% CI	Clinician AUC 95% CI
−1	0.72 ± 0.03	0.55 ± 0.02	4.2*10^−101^	(0.65, 0.78)	(0.5, 0.59)
−3	0.68 ± 0.08	0.63 ± 0.05	4.1*10^−08^	(0.5, 0.83)	(0.52, 0.71)
−5	0.68 ± 0.06	0.51 ± 0.04	9.8*10^−60^	(0.54, 0.79)	(0.44, 0.57)

### 3.5. Patient specific Parkinson Disease network from SPOKE

**Figure 9:**
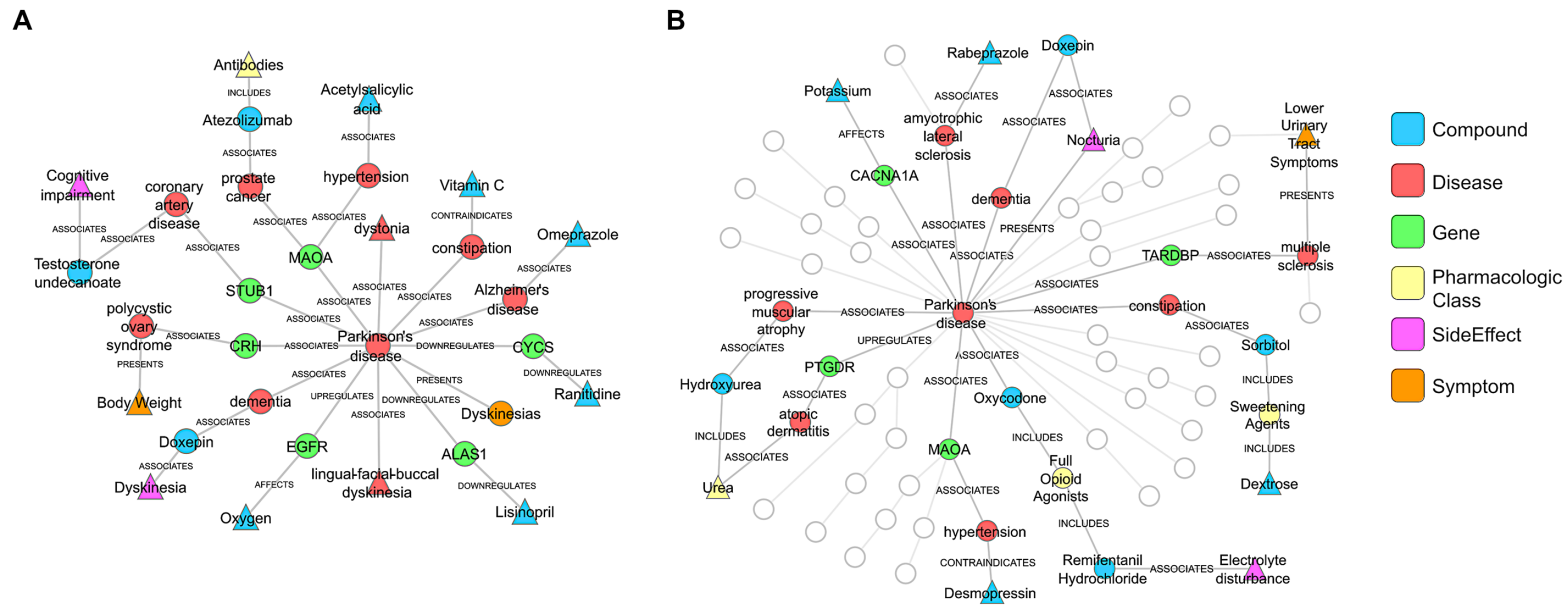
PD patient specific networks from SPOKE **(A)** corresponds to a patient correctly diagnosed by both clinician and SPOKE model. **(B)** corresponds to a patient correctly diagnosed only by SPOKE model. Triangle shaped nodes represent clinical concepts present in patient’s EHR chart and hence the entry points to SPOKE. Circle shaped nodes are the non-entry points. Nodes are connected by edges whose names are shown in the figure. To reduce the complexity of the network **(B)**, certain nodes and edges are grayed out based on manual inspection. Legend shows the color code for each node type in the network.

To further explore the predictive factors underlying the SPOKE-based method, patient specific networks were constructed (Supplementary methods) for a PD patient that was correctly classified by both SPOKE and clinician review (Figure 9A) and another PD patient that was correctly classified by SPOKE but not by clinician review (Figure 9B) in −1 time period. Both patient networks showed enriched connectivity that PD node (center node in both networks) made between biological (for, e.g., genes) and clinical (for, e.g., disease) nodes in SPOKE. These connections could possibly enrich the EHR data of a patient by providing additional biological information relevant to PD through the SPOKEsig vector, thereby enhancing the disease predictivity.

## 4. Discussion

SPOKE-based models (SPOKEsigs) predicted PD diagnosis with moderate accuracy that increased in performance as time to diagnosis approached. The better performance proximate to diagnosis could be in part because of the larger sample size, but also likely due to more PD-relevant information being taken into account. These results potentially reflect the presence of recognizable prodromal symptoms in the years prior to diagnosis that become more numerous and likely more specific as diagnosis nears (1, 8, 9). This interpretation is supported by the feature scores of input nodes where non-motor symptoms (asthenia or generalized weakness, orthostatic intolerance, polyuria, lethargy) are more relevant early and motor symptoms (dysphonia, gait changes, and tremor) arise more proximate to diagnosis (Figures 5D,H,L) as reported in prodromal PD (19).

Using knowledge networks that associate EHR data to other biomedical information, the SPOKE model can access concepts that are not explicitly coded in the EHR such as biological information and hence enriches the clinical data. This enrichment explains the appearance of PD as a relevant node despite the exclusion of the PD diagnostic code from the dataset. This approach also identified molecular and genetic pathways that are highly represented in the pre-diagnostic years of PD and may be used to generate hypotheses of the varying biological processes that occur as prodromal PD progresses (Supplementary Table S7). For instance, OR56A4 – a gene encoding an olfactory receptor – was highly relevant in detecting PD patients even 5 years prior to diagnosis. Impaired olfaction occurs years prior to motor symptoms in PD (20), and the nasopharynx has been proposed as a possible site where environmental toxicants trigger abnormal protein aggregation that then spreads to other brain structures (21). In later years, genes related to mitochondrial dysfunction (APOOL) and immune dysregulation (FGFR1OP2) become more relevant, processes which may underly the cellular damage seen in the substantia nigra during these time periods (22). Genes such as GBA, LRRK2, PINK1, ATP13A2, VPS35, and PARK7 have been reported to have associations with PD (23) and they turned out as critical genes (i.e., high feature importance scores) in this modelling approach for classifying patients in −1 time period. While these associations need rigorous evaluation and testing, they highlight the potential of SPOKE to propose biological targets for biomarkers and therapeutics.

Enrichment of EHR data may explain the higher predictive ability of SPOKE compared to other methods of prediction, such as prediction using raw EHR data (Figure 6), MDS criteria (Figure 7) and clinician review (Figure 8). Notably, both MDS criteria and clinical judgment require more information (e.g., a detailed history, clinical exam, or biologic studies), which may be available in the full medical chart but not in de-identified codes. While SPOKE may not truly be more accurate than these two methods (i.e., MDS and clinician review), the ability of SPOKE to improve predictive accuracy with such sparse information, using much less cost and time than these methods, emphasizes its possible role as a screening tool.

We found that using logistic regression to build the SPOKE classifier was no more accurate than random forest. Previous studies have shown that a random forest model could reduce data overfitting owing to its ensemble architecture and could capture non-linear relationships in the data (24–26). Additionally, random forest models have shown improved interpretability and performance in prior analyses (16, 27). These characteristics could facilitate scalability to larger datasets and ensure that disparate types of data inherent to the SPOKE model are adequately integrated. We therefore chose the random forest model over logistic regression model in this study.

There have been previous efforts to identify people in the pre-diagnostic stages of PD using diagnostic and procedure codes (10, 11). Despite their predictive value, they included data up until the time that PD related codes appeared in the medical chart leaving the possibility that patients already had manifest PD that had not yet been coded. Our inability to validate diagnostic date in our study leaves open a similar possibility, but restricting our model to information that was present years before a diagnostic code and the fact that motor symptoms were less prominent in these time periods suggest we may be identifying people at an earlier stage. Even at earlier stage (i.e., 5 years prior to diagnosis), our model maintained moderate predictive value than other benchmark methods suggesting that enriching EHR data with a biomedical knowledge network, and incorporating a broader scope of data such as diagnosis, medications and laboratory tests, may allow for earlier detection of PD, even before motor symptoms strongly manifest.

There have been previous efforts to create patient representation vectors that were highly predictive (28–32). However, they were abstract latent vectors that cannot be easily interpreted into clinical terms, which may ultimately limit clinician adoption to inform medical decisions (33). A unique value of SPOKE based patient representation is that it is non-abstract and explainable in nature. Each feature in this vector represents a meaningful biomedical concept from the network (Figure 9), making the vector clinically interpretable.

The predictive ability of the SPOKE based model in this project needs to be interpreted in the context of several limitations. Since the present analysis was done on a completely de-identified dataset, diagnosis and index date of diagnosis could not be properly verified. We used stringent criteria to account for this limitation, attempting to avoid common pitfalls such as miscoding or drug-induced parkinsonism. It has been previously reported that PD onset and the first diagnostic code could have a median delay of 1 year (34). To account for this delay, we restricted our analysis to time periods at least 1 year prior to the entry of a diagnostic code. Patients may have received care outside of the UCSF medical system; not having this information available may again have reduced the predictive accuracy of our model. Another limitation is that we have not yet externally validated the SPOKE model. Testing the SPOKE model on a separate dataset will support its generalizability and is an important future direction, though the internal validity demonstrated in this work is encouraging. Finally, some clinical variables in a patient’s EHR would not map to any SPOKE nodes (Supplementary Table S8); expanding SPOKE to include nodes for all EHR variables will be a future goal to enhance the performance of the SPOKE model further.

Despite these limitations, the SPOKE model has the potential to enrich the EHR to identify people at risk of developing PD for more intense clinical evaluation. Future studies can evaluate whether the SPOKE model can distinguish between parkinsonian syndromes (35) - challenging to determine from the EHR alone (36) - or predict outcomes related to PD, such as fractures, falls, or dementia. Additionally, future work will use SPOKE to identify people that can undergo more intensive evaluation to estimate PD risk using clinical and biomarker assessments, such as smell test or imaging of striatal dopamine transporter binding (37). As EHR databases expand to include non-traditional information streams (e.g., sensor data (38), mobile health monitoring (39) and patient reported outcomes (40)), integration with an extensive biomedical knowledge network may not only improve the SPOKE model further, but also provide a crucial strategy to avoid overload (41) and facilitate clinical prediction, further enabling preventive healthcare.

## 5. Conclusion

We showed the application of a biomedical knowledge graph (SPOKE) in enriching the EHR data of patients for an early prediction of PD in a clinically interpretable fashion. This method showed higher predictive performance than other benchmark methods applied to EHR data. We finally showed how biological and clinical information from SPOKE could enhance the PD prediction using patient specific networks. Taken together, the proposed method is an explainable predictive approach for PD detection that could complement clinical decision making.

## Data availability statement

The datasets presented in this article are not readily available due to the sensitive nature of EHR, even in deidentified form. To facilitate the reproducibility and advancement of this research, we have created an API for generating SPOKEsigs alongside a Jupyter notebook with instructions on how to use it, which can be accessed at https://github.com/BaranziniLab/SPOKEsigs. Anyone with access to EHRs can now create SPOKEsigs for their own patient populations and test the concepts presented in this work. SPOKE can be accessed at https://spoke.rbvi.ucsf.edu/neighborhood.html. Requests to access the datasets should be directed to sergio.baranzini@ucsf.edu.

## Ethics statement

Ethical review and approval was not required for the study on human participants in accordance with the local legislation and institutional requirements. Written informed consent from the patients/participants or patients’/participants' legal guardian/next of kin was not required to participate in this study in accordance with the national legislation and the institutional requirements.

## Author contributions

KS gathered data, performed analysis, created figures, and drafted the manuscript. CAN developed methods for the analysis, assisted in the data analysis process, and edited the manuscript. GC assisted in the data analysis process. SMG contributed to study design, assisted with clinical interpretation of the data, and edited the manuscript. SEB assisted with study conception, design, and supervision, and edited the manuscript. EGB assisted with study conception and design, clinical interpretation of the data, and editing of the manuscript. All authors contributed to the article and approved the submitted version.

## Funding

The development of SPOKE and its applications are being funded by grants from the National Science Foundation (NSF_2033569), NIH/NCATS (NIH_NOA_1OT2TR003450), and the UCSF Marcus Program in Precision Medicine Innovation. SEB holds the Heidrich Family and Friends Endowed Chair of Neurology at UCSF. SEB holds the Distinguished Professorship in Neurology I at UCSF.

## Conflict of interest

SEB is cofounder and holds shares in MATE Bioservices, a company that commercializes uses of SPOKE knowledge graph. CAN holds shares of MATE Bioservices.

The remaining authors declare that the research was conducted in the absence of any commercial or financial relationships that could be construed as a potential conflict of interest.

## Publisher’s note

All claims expressed in this article are solely those of the authors and do not necessarily represent those of their affiliated organizations, or those of the publisher, the editors and the reviewers. Any product that may be evaluated in this article, or claim that may be made by its manufacturer, is not guaranteed or endorsed by the publisher.

## Supplementary material

The Supplementary material for this article can be found online at: https://www.frontiersin.org/articles/10.3389/fmed.2023.1081087/full#supplementary-material
