# Supplementary material for: Early detection of Parkinson’s disease through enriching the electronic health record using a biomedical knowledge graph
Source: Front Med (Lausanne). 2023 May 12;10:1081087. doi: 10.3389/fmed.2023.1081087 (PMC10217780; doi:10.3389/fmed.2023.1081087)
Supplement: Supplementary file 1 [file Data_Sheet_1.docx]

***Supplementary Material***

**1. Supplementary Methods**

**Patient selection**

We used de-identified Electronic Health Record (EHR) data of patients who visited UCSF Medical Center between 2010 and 2020. For this study, we selected three tables from the EHR: conditions (International Classification of Diseases (ICD) and Systematized Nomenclature of Medicine-Clinical Terms (SNOMED-CT) diagnostic codes) from either inpatient or outpatient settings, measurements (laboratory test results, Logical Observation Identifiers Names and Codes (LOINC)) and drugs (medications prescribed, RxNorm codes). The EHR clinical concepts were made available in Observational Medical Outcomes Partnership (OMOP) format, a model that transforms data contained within disparate observational databases into a common format (1).

PD patients were selected based on the presence of diagnostic codes indicative of PD (Supplementary Table 1) with at least two entries separated by a period of six months. This temporal separation was required to avoid coding errors or diagnoses solely entered to justify a diagnostic test to rule out PD. To avoid inclusion of patients with neuroleptic-induced parkinsonism, a common misdiagnosis, patients on neuroleptic medications (Supplementary Table 2) within six months before their first PD diagnosis were excluded. Non-PD individuals were selected as those with no PD diagnostic code present in their EHR and who had at least six months of information present, to temporally align them with the PD population. Finally, we restricted the entire population to 40 years of age or older, to minimize the inclusion of people with rare genetic forms of PD who may have patterns of onset different than sporadic PD. Implementing this age criteria also avoids overrepresentation of younger controls, which would lead to conditions associated with aging appearing to be associated with PD development. The index date for PD was defined as the first entry of a PD code or, for those started on medications for PD (Supplementary Table 3) prior to the appearance of the EHR code, the date this medication was started. Index date for non-PD was defined as six months prior to the last visit, to align with the minimal six months follow up period required for the PD group after their first diagnosis. In order to build a classifier that would identify people at risk of PD in the general population, we trained the model for each time period using a case:control ratio based on the age-adjusted prevalence of PD, i.e. 572:100,000 among people of age 45 and over (2), which closely matches the age threshold in this study.

After selecting PD and non-PD populations, we categorized their EHR data into three pre-diagnostic time periods. EHR data present one, three and five years prior to their index date were grouped into -1, -3 and -5 periods respectively. Since these periods denoted the pre-diagnosis phase, no PD diagnosis codes were present in the EHR data used to train and test the SPOKE model.

**Creation of patient embedding vectors**

We adopted a knowledge graph method to create machine-readable representations of patients and called it SPOKEsigs, short for SPOKE signatures (3). SPOKE is a knowledge graph which integrates information from over 30 different databases, consisting of more than 3 million nodes of 16 types and more than 16 million edges of 32 types(3). SPOKEsigs are linear combinations of vectors known as Propagated SPOKE Entry Vectors (PSEVs) (3, 4). These vectors are generated by embedding and diffusing (by means of a random walker) EHR data of a patient cohort on the SPOKE knowledge graph(4). Therefore, a PSEV represents a clinical concept (e.g. Parkinson’s disease, tremor) in terms of the SPOKE knowledge graph. Each dimension of a PSEV corresponds to a node in SPOKE. The value of a dimension depicts the importance of that SPOKE node with respect to the clinical concept represented by that PSEV.

Before invoking prediction model (i.e. training and testing), SPOKEsigs undergo various transformations such as rank normalization (i.e. ranking the values of each dimension) followed by z-score normalization and finally node specific rank normalization (i.e. ranking the values based on node type to capture the node heterogeneity of SPOKE)(3). Since a majority of nodes in the SPOKEsig vector were of Compound node type (73.7%), only high variance Compound nodes were taken into account (making “Compound” count to be same as the second largest SPOKE node type which is “Protein”). This brings down the dimension of the SPOKEsig vector from 389,297 to 136,364.

**Identifying top feature nodes in patient classification**

Random forest classifier is an explainable model, which means that one can check upon the top input features that the model relied upon for classification. Feature importance intuitively conveys how important a feature was in splitting data into separate classes (5). To compute input feature scores corresponding to a time period, we trained hundred random forest models on different training datasets of that year. Feature importance scores were then averaged across these hundred models. These averaged feature scores were then used to assess the top feature nodes from the input data in classifying patients into PD vs non-PD classes in a time period.

**Comparison with logistic regression model**

We compared the performance of the random forest classifier with a logistic regression model to account for any algorithm-specific differences in predicting PD using SPOKEsig vectors. We ensured that the same train and test patient datasets used for random forest were utilized for logistic regression. This allowed us to compare the performance of both classifiers in predicting PD during the pre-diagnostic time period of -1 year. The logistic regression model was built using TensorFlow and trained using a data generator class that pulls batches of patient SPOKEsig vectors in a parallelized fashion to optimize the memory and training time. We used a batch size of 256 and shuffled the indices of training data at the end of each epoch of model training. This ensured that the model was exposed to a different sequence of batches of training data in each epoch, which would increase the robustness of the model. The model was trained using Adam (adaptive moment estimation) optimizer on binary cross-entropy loss function for 100 epochs. While training, PD samples were weighted more heavily than non-PD samples based on their distribution in the training data. This was done to take care of the class-imbalance in the dataset. To avoid data overfitting and to enhance model generalization, we used early stopping criteria while training the model by monitoring the AUC score on a validation dataset (which was split from the training data in the ratio of 80[training]:20[validation]). After training, the logistic regression model was tested on the same patient test set used for random forest classifier.

**Comparison with raw EHR data**

For comparative analysis, we did PD prediction using raw EHR data (i.e. without SPOKE enrichment). Using the same patient population, we created a binary representation vector using the EHR chart from each time period. Each element in that vector corresponded to an EHR concept; we assigned a value of 1 if that concept was present in that patient’s EHR chart for that time period and a value of 0 if the concept was absent. The dimension of each vector corresponded to the total unique EHR concepts available for both PD and non-PD populations in each time period. For a fair comparison with SPOKE, we restricted EHR concepts to those mappable to SPOKE nodes (Supplementary Table 8), resulting in 31479, 26448 and 19880 binary vector dimensions for time periods -1, -3 and -5 years respectively. In each time period, 80% of the data was used to train a random forest classifier which was then tested on the remaining 20% of the data. Classifier performance was analyzed by bootstrapping the predictions (100 times) on the test dataset which resulted in an AUC distribution. This process was repeated for each time period. These distributions were then compared (using t-test) with corresponding AUC distributions of SPOKE based random forest classifiers, generated using the same test patients of a time period.

**Comparison with MDS criteria**

SPOKE based prediction results were compared with analysis of EHR data according to the proposed research criteria for prodromal Parkinson’s disease developed by the International Parkinson and Movement Disorder Society (MDS) (6, 7). The MDS method estimates a likelihood ratio for future PD diagnosis based on the presence or absence of numerous risk and prodromal markers that are supported by the literature. Out of the 23 risk and prodromal markers listed in the most recent criteria (7), we were able to map 17 of them to OMOP EHR codes (Supplementary Table 6). These codes were then weighted according to their reported likelihood ratios in the criteria.

We used the likelihood ratio and prior probability to compute the posterior probability for prodromal PD (7). These posterior probabilities were further used for AUC performance analysis using the bootstrap method. This generated an AUC distribution for the MDS analysis for each time period. We used t-test to compare these distributions to the corresponding AUC distributions of SPOKE based random forest classifiers, generated using the same MDS patient samples of a time period.

**Comparison with clinician review**

SPOKE based prediction results were compared with the review of de-identified EHR data by a movement disorders neurologist specialized in the diagnosis and therapeutics of PD and other movement related disorders. The neurologist was provided with the pre-diagnosis EHR data discretized into -1, -3 and -5 time periods, though the specific time period information associated with the data was not disclosed. Each time period had 100 unique patients. Patient labels (i.e. PD or non-PD) and prevalence ratio of PD:non-PD in the dataset were not disclosed to the clinician, and patients were sorted randomly. The neurologist was provided with those EHR data that were also inputs to the SPOKE based classifier approach. In that way, we made sure that both clinician and classifier evaluated the same set of clinical concepts. The neurologist then classified cases as either prodromal PD or not.

AUC analysis using the bootstrap approach was then applied to neurologist’s predictions and resulted in an AUC distribution for each time period. We used t-test to compare these distributions across time periods with the corresponding AUC distributions of SPOKE based random forest classifiers, generated using the same patient samples provided to the neurologist.

**Patient specific PD network from SPOKE**

To demonstrate biologically significant connections on a patient level, connections to the PD node were extracted from SPOKE for PD patients in the -1 time period. From this cohort, we randomly selected two PD patients such as: (i) patient that was correctly diagnosed by both neurologist and SPOKE model and (ii) patient that was correctly diagnosed only by SPOKE model, to demonstrate the utility of SPOKE above clinician review. Each patient specific network was created by concatenating all the shortest paths between EHR concepts of a patient (those mappable to SPOKE nodes) and PD node in SPOKE. This network was visualized using Cytoscape, an open-source bioinformatics software platform for visualizing networks.

**References**

1. Stang PE, Ryan PB, Racoosin JA, Overhage JM, Hartzema AG, Reich C, et al. Advancing the Science for Active Surveillance: Rationale and Design for the Observational Medical Outcomes Partnership. *Ann Intern Med* (2010) 153(9):600-6. doi: 10.7326/0003-4819-153-9-201011020-00010.

2. Marras C, Beck JC, Bower JH, Roberts E, Ritz B, Ross GW, et al. Prevalence of Parkinson's Disease across North America. *NPJ Parkinsons Dis* (2018) 4:21. doi: 10.1038/s41531-018-0058-0.

3. Nelson CA, Bove R, Butte AJ, Baranzini SE. Embedding Electronic Health Records onto a Knowledge Network Recognizes Prodromal Features of Multiple Sclerosis and Predicts Diagnosis. *J Am Med Inform Assoc* (2022) 29(3):424-34. doi: 10.1093/jamia/ocab270.

4. Nelson CA, Butte AJ, Baranzini SE. Integrating Biomedical Research and Electronic Health Records to Create Knowledge-Based Biologically Meaningful Machine-Readable Embeddings. *Nat Commun* (2019) 10(1):3045. doi: 10.1038/s41467-019-11069-0.

5. Strobl C, Boulesteix A-L, Zeileis A, Hothorn T. Bias in Random Forest Variable Importance Measures: Illustrations, Sources and a Solution. *BMC Bioinformatics* (2007) 8:25. doi: 10.1186/1471-2105-8-25.

6. Berg D, Postuma RB, Adler CH, Bloem BR, Chan P, Dubois B, et al. Mds Research Criteria for Prodromal Parkinson's Disease. *Mov Disord* (2015) 30(12):1600-11. doi: 10.1002/mds.26431.

7. Heinzel S, Berg D, Gasser T, Chen H, Yao C, Postuma RB, et al. Update of the Mds Research Criteria for Prodromal Parkinson's Disease. *Mov Disord* (2019) 34(10):1464-70. doi: 10.1002/mds.27802.

**2. Supplementary Figures**

**
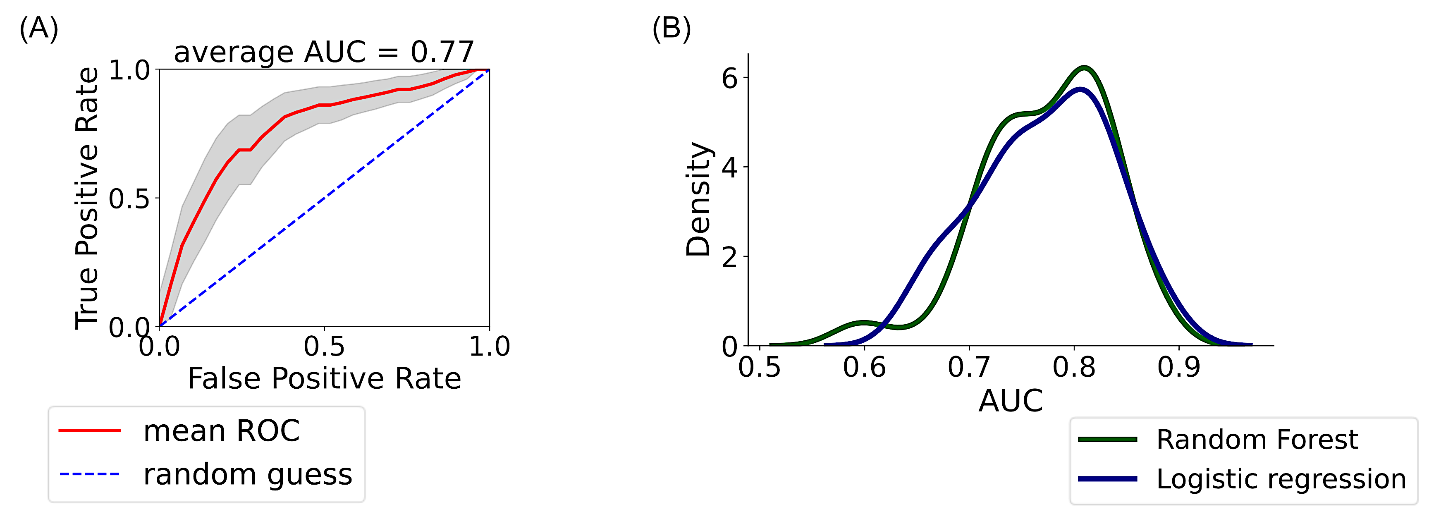
**

**Supplementary Figure 1.** PD prediction using logistic regression at -1 year pre-diagnostic time period **(A)** shows the ROC performance curve of logistic regression model with red curve depicting the average ROC curve, gray shade showing ± standard deviation, and blue dotted line representing the random guess curve. Average AUC of ROC curve is 0.77 as shown at the top of the box. **(B)** shows the AUC distributions of random forest (green) and logistic regression (blue) on the same test data at -1 year pre-diagnostic time period.

**3. Supplementary Tables**

**Supplementary Table 1**: Diagnostic codes indicative of PD used for patient selection

| **Concept name** | **Concept code** | **Vocabulary** |
| --- | --- | --- |
| Parkinson's disease | 49049000 | SNOMED |
| Parkinson's disease | 332 | ICD9CM |
| Parkinson's disease | G20 | ICD10CM |
| Cerebral degeneration due to Parkinson's disease | 438513002 | SNOMED |
| Dementia associated with Parkinson's Disease | 425390006 | SNOMED |
| Sporadic Parkinson disease | 724761004 | SNOMED |
| Orthostatic hypotension co-occurrent and due to Parkinson's disease | 718685006 | SNOMED |
| Psychosis co-occurrent and due to Parkinson's disease | 719717006 | SNOMED |
| Dopamine transporter deficiency syndrome | 722763000 | SNOMED |
| Young onset Parkinson disease | 715345007 | SNOMED |
| Autosomal dominant late onset Parkinson disease | 716662004 | SNOMED |

**Supplementary Table 2**: Medications used to exclude patients during PD patient selection

| **Generic name** | **Brand name** |
| --- | --- |
| Aripiprazole | Abilify, Abilify Maintena |
| Asenapine | Saphris, Sycrest |
| Cariprazine | Vraylar |
| Clozapine | Clozaril |
| Brexpiprazole | Rexulti |
| Chlorpromazine | Largactil, Thorazine |
| Haloperidol | Haldol, Peridol |
| Lurasidone | Latuda |
| Olanzapine | Zyprexa |
| Paliperidone | Invega, Xeplion |
| Pimozide | Orap |
| Quetiapine | Seroquel |
| Risperidone | Risperdal, Rispolept |
| Ziprasidone | Geodon, Zeldox |

**Supplementary Table 3**: Medications that change the first diagnosis date of PD patients

| **Generic name** | **Brand name** |
| --- | --- |
| Symmetrel | Amantadine |
| Gocovri | Amantadine, extended release |
| Apokyn | Apomorphine |
| Apomorphine sublingual film | Kynmobi |
| Cogentin | Benztropine |
| Lodosyn | Carbidopa |
| Stalevo | Carbidopa, levodopa, and entacapone |
| Duopa or Duodopa | Carbidopa-levodopa intestinal gel |
| Sinemet | Carbidopa-levodopa oral |
| Parcopa | Carbidopa-levodopa oral |
| Rytary or Numient | Carbidopa-levodopa oral, extended release |
| Sinemet CR | Carbidopa-levodopa oral, extended release |
| Carbidopa / Levodopa and Entacapone Intestinal Gel | Lecigon |
| Carbidopa / Levodopa Inhalation Powder | Inbrija |
| Comtan | Entacapone |
| Madopar | Levodopa-benserazide |
| Levodopa / Benserazide Controlled Release | Madopar CR, Madopar HBS, or Prolopa CR |
| Levodopa / Benserazide Dispersible | Madopar Rapid |
| Mirapex, Mirapexin or Sifrol | Pramipexole |
| Mirapex ER or Sifrol ER | Pramipexole |
| Azilect | Rasagiline |
| Adartel | Ropinirole |
| Requip | Ropinirole |
| Ropinirole | Requip or Adartel |
| Requip XL | Ropinirole |
| Ropinirole Extended Release | Requip XL |
| Neupro | Rotigotine |
| Xadago | Safinamide |
| Eldepryl | Selegiline |
| Deprenyl | Selegiline |
| Emasm | Selegiline |
| Zelapar | Selegiline |
| Artane | Trihexyphenidyl |
| Apo-Trihex | Trihexyphenidyl |
| Trihexyphenidyl | Artane or Apo-Trihex |
| Tasmar | Tolcapone |
| Ethopropazine | Parsitan or Parsidan or Profenamine or Parsidol, or Parkin |
| Istradefylline | Nourianz or Nouriast |
| Mucuna Pruriens | Mucuna Pruriens |
| Opicapone | Ongentys |

**Supplementary Table 4**: Patient demographics

| Demographics | PD | non-PD |
| --- | --- | --- |
| Total patients | 3,004 | 457,197 |
| Age (μ±σ) | 68±9.9 | 60±12.6 |
| Sex | Male: 63.75%  Female: 36.22%  Other: 0.03% | Male: 46.3%  Female: 53.6%  Other: 0.1% |
| Number of patients in year -1 | 437 | 168,499 |
| Number of patients in year -3 | 237 | 107,194 |
| Number of patients in year -5 | 108 | 62,590 |

**Supplementary Table 5**: Patient demographics in each pre-diagnosis time period

| **Demographics** | **Year -1** | | **Year -3** | | **Year -5** | |
| --- | --- | --- | --- | --- | --- | --- |
|  | **PD** | **non-PD** | **PD** | **non-PD** | **PD** | **non-PD** |
| Age (μ±σ) | 71±10 | 61±12.9 | 71±9.7 | 62±13 | 69±9.9 | 62±12.9 |
| Sex | Male:  62%  Female: 38% | Male:  44.66%  Female:  55.32%  Other:  0.02% | Male: 57.4%  Female: 42.6% | Male:  43.64%  Female:  56.35%  Other:  0.01% | Male:  56.5 %  Female:  43.5% | Male:  42.28%  Female:  57.71%  Other:  0.01% |
| Number of patients | 437 | 168499 | 237 | 107194 | 108 | 62590 |

**Supplementary Table 6**: MDS markers used in this study

| **Sl. no.** | **MDS marker** |
| --- | --- |
| 1 | Regular pesticide exposure |
| 2 | Occupational solvent exposure |
| 3 | Nonuse of caffeine |
| 4 | Diabetes mellitus (type II) |
| 5 | Low plasma urate levels |
| 6 | PSG-proven RBD |
| 7 | Constipation |
| 8 | Excessive daytime somnolence |
| 9 | Orthostatic hypotension (OH) |
| 10 | Erectile dysfunction |
| 11 | Urinary dysfunction |
| 12 | Depression |
| 13 | Anxiety |
| 14 | Global cognitive deficit |
| 15 | Olfactory loss |
| 16 | Male sex |
| 17 | Symptomatic OH |

**Supplementary Table 7**: Top biological nodes in SPOKE that drive classifier PD predictions in each year

| **Sl. no.** | **Year** | **Biological Node Type** | **Node Name** | **Rank** |
| --- | --- | --- | --- | --- |
| 1 | -1 | Gene | USP40 | 1 |
| 2 | -1 | Gene | ATP1B4 | 2 |
| 3 | -1 | Gene | ZNF25 | 3 |
| 4 | -1 | Gene | CCDC125 | 4 |
| 5 | -1 | Gene | PINLYP | 5 |
| 6 | -1 | BiologicalProcess | granulocyte colony-stimulating factor signaling pathway | 1 |
| 7 | -1 | BiologicalProcess | regulation of dopamine biosynthetic process | 2 |
| 8 | -1 | BiologicalProcess | cell growth | 3 |
| 9 | -1 | BiologicalProcess | positive regulation of interleukin-23 production | 4 |
| 10 | -1 | BiologicalProcess | response to dopamine | 5 |
| 11 | -1 | MolecularFunction | type I interferon receptor activity | 1 |
| 12 | -1 | MolecularFunction | ATPase-coupled cation transmembrane transporter activity | 2 |
| 13 | -1 | MolecularFunction | dephospho-CoA kinase activity | 3 |
| 14 | -1 | MolecularFunction | P-type divalent copper transporter activity | 4 |
| 15 | -1 | MolecularFunction | primary active transmembrane transporter activity | 5 |
| 16 | -3 | Gene | RAB1A | 1 |
| 17 | -3 | Gene | APOOL | 2 |
| 18 | -3 | Gene | ABO | 3 |
| 19 | -3 | Gene | FGFR1OP2 | 4 |
| 20 | -3 | Gene | KRTAP5-9 | 5 |
| 21 | -3 | BiologicalProcess | mature B cell differentiation involved in immune response | 1 |
| 22 | -3 | BiologicalProcess | thymidine metabolic process | 2 |
| 23 | -3 | BiologicalProcess | cell volume homeostasis | 3 |
| 24 | -3 | BiologicalProcess | macromolecule glycosylation | 4 |
| 25 | -3 | BiologicalProcess | regulation of intrinsic apoptotic signaling pathway in response to hydrogen peroxide | 5 |
| 26 | -3 | MolecularFunction | G protein-coupled chemoattractant receptor activity | 1 |
| 27 | -3 | MolecularFunction | aryl sulfotransferase activity | 2 |
| 28 | -3 | MolecularFunction | protein kinase C inhibitor activity | 3 |
| 29 | -3 | MolecularFunction | protein transmembrane transporter activity | 4 |
| 30 | -3 | MolecularFunction | immunoglobulin receptor activity | 5 |
| 31 | -5 | Gene | OR56A4 | 1 |
| 32 | -5 | Gene | RNF19A | 2 |
| 33 | -5 | Gene | FCGR2C | 3 |
| 34 | -5 | Gene | ZC4H2 | 4 |
| 35 | -5 | Gene | TAS2R19 | 5 |
| 36 | -5 | BiologicalProcess | regulation of lysosomal protein catabolic process | 1 |
| 37 | -5 | BiologicalProcess | polyamine metabolic process | 2 |
| 38 | -5 | BiologicalProcess | positive regulation of hematopoietic progenitor cell differentiation | 3 |
| 39 | -5 | BiologicalProcess | negative regulation of mitotic sister chromatid separation | 4 |
| 40 | -5 | BiologicalProcess | DNA damage induced protein phosphorylation | 5 |
| 41 | -5 | MolecularFunction | acetyl-CoA binding | 1 |
| 42 | -5 | MolecularFunction | calcium-dependent protein kinase inhibitor activity | 2 |
| 43 | -5 | MolecularFunction | structural constituent of postsynaptic intermediate filament cytoskeleton | 3 |
| 44 | -5 | MolecularFunction | interleukin-8 receptor binding | 4 |
| 45 | -5 | MolecularFunction | nucleoside kinase activity | 5 |

**Supplementary Table 8**: EHR coverage in SPOKE for each time period

| **Year** | **Condition** | **Medication** | **Lab test** |
| --- | --- | --- | --- |
| -1 | 74% | 60% | 30% |
| -3 | 74% | 60% | 31% |
| -5 | 74% | 59% | 31% |
